# Sensitive Planar Microwave Diode on the Base of Ternary Al*_x_*Ga_1-*x*_As Semiconductor Compound

**DOI:** 10.3390/s21134487

**Published:** 2021-06-30

**Authors:** Maksimas Anbinderis, Steponas Ašmontas, Aurimas Čerškus, Jonas Gradauskas, Andžej Lučun, Aldis Šilėnas, Algirdas Sužiedėlis

**Affiliations:** 1 Center for Physical Sciences and Technology, Savanorių Ave. 231, 02300 Vilnius, Lithuania; maksimas.anbinderis@ftmc.lt (M.A.); steponas.asmontas@ftmc.lt (S.A.); aurimas.cerskus@ftmc.lt (A.Č.); jonas.gradauskas@ftmc.lt (J.G.); andzej.lucun@ftmc.lt (A.L.); aldis.silenas@ftmc.lt (A.Š.); 2 Department of Physics, Vilnius Gediminas Technical University, Saulėtekio Ave. 11, 10223 Vilnius, Lithuania

**Keywords:** planar diode, microwave sensor, voltage responsivity, aluminum gallium arsenide

## Abstract

The article presents the results of experimental studies of the dc and high-frequency electrical characteristics of planar microwave diodes that are fabricated on the base of the *n*-Al*_x_*Ga_1-*x*_As layer (*x* = 0, 0.15 or 0.3), epitaxially grown on a semi-insulating GaAs substrate. The diodes can serve as reliable and inexpensive sensors of microwave radiation in the millimeter wavelength range; they sense electromagnetic radiation directly, without any external bias voltage at room temperature. The investigation revealed a strong dependence of the detection properties of the microwave diodes on AlAs mole fraction *x*: in the K_a_ microwave frequency range, the median value of voltage responsivity is several volts per watt in the case of GaAs-based diodes (*x* = 0), and it substantially increases, reaching hundreds of volts per watt at higher *x* values. Also, a model enabling us to forecast the responsivity of the sensor in other frequency ranges is proposed.

## 1. Introduction

Electromagnetic radiation in the millimeter wavelength range attracts the attention of scientists and engineers due to its numerous possible applications in modern fields of technology, such as the imaging of concealed objects [1,2,3], inspection of material homogeneity [4,5], and arranging broadband cellular communication networks [6,7]. Successive use of the millimeter waves in frequency-modulated continuous wave (FMCW) radar technology, in conjunction with small-sized MIMO (multiple-input multiple-output) antenna arrays, gives possibility for the precise scanning of nearby situated various objects. Texas Instruments Inc. produces low-cost automotive and industrial millimeter wave sensors for radaring either automotive vehicles or smaller industrial objects [8,9]. The fast development of such technologies requires novel concepts of electromagnetic radiation sensors. Some millimeter wave sensors require bulky cryogenic cooling equipment (high-T_c_ Josephson junction detector [10] or Y–Ba–Cu–O microbolometer [11]). Some of them are quite complex, such as a Golay cell, which, on the other hand, demonstrates its advantage of high-voltage responsivity at room temperature and the ability to sense electromagnetic radiation in a wide spectral range, from ultraviolet light up to K_a_ microwave (MW) frequencies [12]. Backward tunnel diodes [13], Si CMOS field-effect transistors [14], and a big variety of microwave diodes on the base of Schottky junction [15] are also known as room-temperature sensors of the millimeter waves. The most popular of them are zero-biased low-barrier Schottky diodes. These are favorable in small-signal square law and envelope detection [16,17], and thus are useful in the application of wireless electromagnetic communication. High-voltage responsivity, low-noise characteristics, operation at room temperature, wide bandwidth, competent reliability, and good repeatability make the Schottky diodes unquestioned leaders in the square detection race. The Schottky diodes have reached their commercial maturity, allowing the purchase of these diodes in the market [18]. However, the fundamental concept of a Schottky junction requires it to be formed on a semiconductor surface that makes the diode’s properties sensitive to the manufacturing conditions and vulnerable to the impact of environment. Therefore, scientists all around the world are looking for new concepts of electromagnetic radiation sensors having detection processes situated in the bulk of a device. More than fifty years ago, promising microwave diodes operating on the basis of hot carrier phenomena in semiconductors, under the action of a nonhomogeneous electric field, were developed [19]. Further improvements in these point-contact diodes turned into the planar bow-tie design of the diodes, successfully sensing electromagnetic radiation from the microwaves to the infrared, including the terahertz frequency range [20,21,22]. These bow-tie diodes were successfully used for heterodyne and spectroscopic terahertz imaging and sensing [22,23]. Low-voltage responsivity was the main drawback of the bow-tie diode; it was increased by means of partial gating of the active two-dimensional electron gas layer in the vicinity of the diode’s contacts [24]. Another kind of microwave diodes having the active region situated in the bulk of a device is the heterojunction diode. In the sense of increasing their voltage responsivity, the point-contact [25] and planar [26] heterojunction diodes revealed themselves as counterparts of the hot carrier microwave diodes that are worth attention. As for commercial application, the microwave sensors must be not only sensitive and fast, but also reliable and inexpensive. We have proposed a simple design of an inexpensive planar dual microwave diode, fabricated on the base of low-resistivity gallium arsenide (GaAs) substrate [27]. The diodes were suitable both to sense microwave continuous wave signals and to measure pulsed microwave power in the nanosecond time scale [28]. However, such fast diodes, which can detect pulses of nanosecond duration, suffered from low-voltage responsivity, while more sensitive ones had high electrical resistance in the MΩ range. Thus, the main issue of this paper is increasing the voltage responsivity of the microwave radiation sensor on the base of the planar dual microwave diode, and narrowing the spread of its electrical parameters by means of its fabrication on the base of epitaxial aluminum gallium arsenide *n*-Al*_x_*Ga_1-*x*_As layer, grown on a semi-insulating GaAs substrate.

The paper describes a detailed procedure of the sample fabrication, as well as naming the measurement technique used and the research methodology applied (Section 2). Section 3 presents the main results of the investigation and discussion concerning the following: (*i*) statistical data of three batches of planar microwave diodes; (*ii*) dependence of electrical parameters of the representative diodes on illumination and temperature; (*iii*) fitting experimental data of dc and high-frequency parameters of the sensors using phenomenological theory; and (*iv*) evaluation of the ability of the planar microwave diode to withstand the impact of high-power microwave radiation.

## 2. Samples and Measurement Technique

The active region of the microwave diode was epitaxial *n*-type Al*_x_*Ga_1-*x*_As layer of sub-micrometric thickness grown onto a semi-insulating GaAs (100) substrate (350 AGChP-I-26a (100) 0°30′, Ø26 mm, Plant of Pure Metals, Svitlovodsk, Ukraine) by means of liquid phase epitaxy technique in the quartz tube furnace “SDO-125/3” (Termotron, Bryansk, USSR). Epitaxial layers were grown using the supercooled growth method in “wiping-less” horizontal graphite sliding boat. Initial supercooling was performed at 6 °C below the melting point of Ga–GaAs mixture. The growth temperature interval was (803–802) °C with 0.5 °C/min cooling speed. The epitaxial layers were not doped intentionally. AlAs mole fraction *x* in the Al*_x_*Ga_1-*x*_As semiconductor was chosen as *x* = 0; 0.15; and 0.3. Optical microscope was used to measure the thickness of the grown layers by taking a cross-sectional view of the structure fraction. Electrical characteristics of the layers were measured using Van der Pauw technique at room temperature. The main parameters of the layers are presented in Table 1.

**Figure 1:**
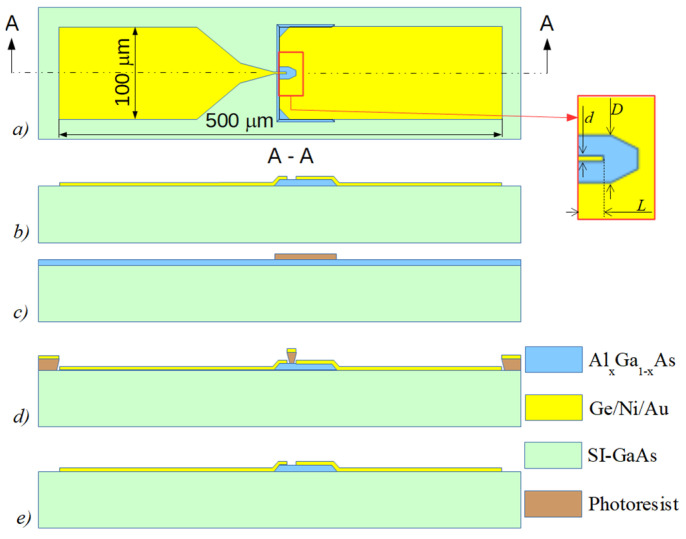
Schematic top (**a**) and cross-sectional (**b**) views of the planar microwave diode. Successive fabrication steps of the diode are shown in parts (**c***–***e**).

**Figure 2:**
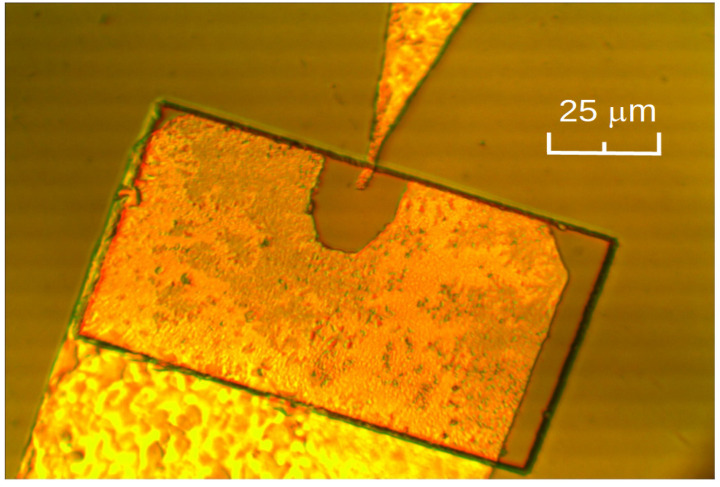
Micrograph of the planar microwave diode on semi-insulating GaAs.

Schematic view of the planar microwave diode is presented in Figure 1a, and its cross-sectional view is shown in Figure 1b. Simple fabrication procedure of the diode is sketched in Figure 1c–e. Before the sample processing, the grown wafers were rinsed in acetone and methanol. First, photolithography was performed using positive Shipley S1805 photoresist to form rectangular 105 × 60 μm^2^ AlGaAs mesas (see Figure 1c). Phosphorus-based etchant of special composition, H_3_O_4_:H_2_O_2_:H_2_O = 1:4:45 [29], was chosen on purpose, creating mesa with flat slopes; this is important to protect the metal terminal from cracking on the mesa’s slope. The etched mesa’s slope was controlled using “Dektak 6M” (Veeco Metrology LLC, Plainview, NY, USA) stylus profilometer. The angle of the slope ranged between 21° and 24° independently of orientation of the mesas, with respect to crystallographic directions of the semiconductor substrate. Second, photolithography was carried out using image reversal MicroChemicals AZ 5214E photoresist to open the windows for the deposition of metallic contacts. The width *d* of the tip of the left contact (see Figure 1a) varied in the range of 1–4 μm in different MW diodes. Metallic contacts of the diodes were fabricated by means of thermal successive evaporation of Ni:Au:Ge:Ni:Au metals of respective thickness 5:200:100:75:100 nm onto a photo-resistive mask using “VAKSIS PVD Vapor-5S_Th” (Vaksis, Ankara, Turkey) (Figure 1d). Composition of Ge–Ni–Au-based metals was chosen because it assures good Ohmic contacts to *n*-GaAs and AlGaAs [30]. The contact patterns were formed using the lift-off technique with consequent annealing of the structures at 430 °C in hydrogen atmosphere in the tube furnace (Figure 1e). At this stage, it is worth noting, the fabrication procedure of the planar MW diodes situated on the semi-insulating substrate was completed, and such samples were handy for the measurements on a probe station. In contrast, the dual MW diodes that were based on low-resistivity GaAs substrate [27] additionally needed to be transferred onto a dielectric polyimide film in order to avoid electrical shunting through the low-resistivity GaAs substrate. Micrographic view of the finished planar MW diode on semi-insulating substrate is presented in Figure 2.

Probe stations were used to measure both dc and high-frequency parameters of the microwave diodes. The choice of these instruments was advantageous in collecting more informative statistical data of the diodes. The current–voltage (IV) characteristics were measured using Süss Micro Tec probe station EP6 (FormFactor, Inc., Livermore, CA, USA) and Agilent E5270B precision measurement equipment (Agilent Technologies, Inc., Santa Clara, CA, USA). Voltage–power (VP) characteristics of the diodes in the K_a_ frequency range were measured using Cascade Microtech (FormFactor, Inc., Livermore, CA, USA) high-frequency probe station, and probes ACP40-A-GS-250 were used to connect the investigated MW diode to the measurement device. SHF BT45 broadband bias tee separated the detected dc voltage signal from the microwave signal. Application of the probe stations gave additional options in the investigation of electrical properties of the planar microwave diodes, i.e., we could measure the characteristics depending on light illumination and temperature. Most measurements were carried out at room temperature, but use of commercially available Peltier modulus let us perform both dc and high-frequency measurements up to 80 °C temperature.

The sequence of the study of the fabricated planar MW diodes was as follows. First, separate MW diodes were tested in their array on the semiconductor substrate. Electrical resistance at zero applied voltage, which was evaluated from the measured IV characteristics. Then the voltage responsivity of the diodes was measured at *f* = 30 GHz frequency. The level of the microwave power was chosen to be in the range where the detected voltage depended linearly on the power. Median values of the voltage responsivity and electrical resistance of the diodes were calculated after statistical processing of the measured data. For further, more detailed investigation, the diodes having electrical parameters close to the median values were selected from all three groups of different AlAs mole fraction-containing samples (having *x* = 0, 0.15 and 0.3). To find out the influence of illumination on the electrical parameters of the MW diodes, the IV and VP (at *f* = 30 GHz) characteristics were measured in the dark and under photo lamp Eiko EKE21V150W (color temperature 3240 K) light illumination. Frequency dependence of the voltage responsivity was measured within the K_a_ frequency range. The responsivity of the diodes in wider frequency range was performed by fitting the experimental responsivity data and using the phenomenological equation of the dependence of voltage responsivity on non-linearity parameters of IV characteristics of the diodes. The hardiness of the diodes to withstand maximum power of the MW radiation was evaluated using the value of the highest measured applied voltage at which the IV characteristic still did not begin changing.

## 3. Results and Discussion

**Figure A1:**
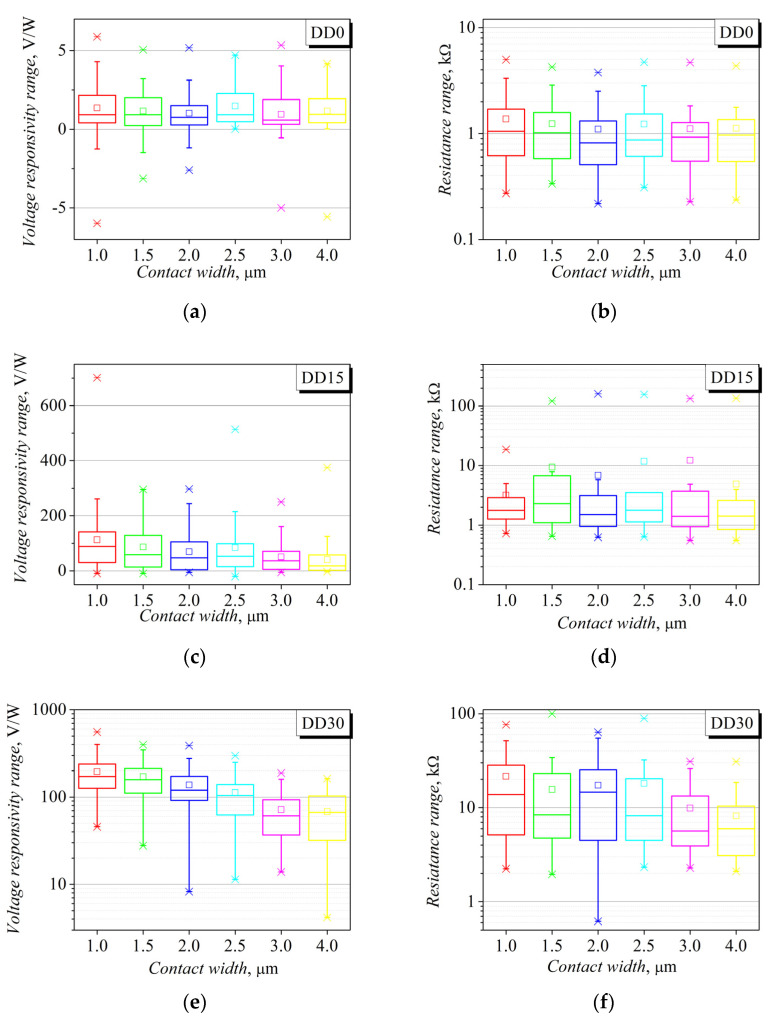
Box-charts of the voltage responsivity (**a**,**c**,**e**) and electrical resistance (**b**,**d**,**f**) of the planar microwave diodes DD0 (**a**,**b**); DD15 (**c**,**d**); DD30 (**e**,**f**)with different width of the small contact.

Three batches of different kinds of the diodes, DD0, DD15 and DD30, were named according to the percentage of AlAs mole fraction in the Al*_x_*Ga_1-*x*_As semiconductor compound. The fabricated diodes also had different widths *d* of the small area contact, as follows: 1.0, 1.5, 2.0, 2.5, 3.0, and 4.0 micrometers. Each batch contained 480 diodes. The output of the operative diodes in the DD0, DD15 and DD30 batches was 83%, 78% and 46%, respectively. The two-fold decrease in the number of the operative diodes in the case of DD30 can be explained by a significant increase in the diode’s resistance when the molar fraction of AlAs exceeds 20%, and when the deep recombination centers (DX centers) in the AlGaAs compound begin to appear [31]. A negative potential of the detected voltage was predominantly measured on the small area contact (left contact in Figure 1 and Figure 2). Let us label this polarity detected voltage as a Schottky voltage. However, the voltage of opposite polarity was detected across some operative DD0 and DD15 diodes, and it amounted to 7% and 4%, respectively. Let us call this detected voltage a TEMF voltage, because its polarity is inherent to the thermoelectromotive force of the hot carriers measured across the microwave diodes having perfect Ohmic contacts [19]. The box charts of the statistical results are presented in Figure A1 of Appendix A. The median value of the voltage responsivity of the diodes can be approximated by its linear dependence on the width of the contact *d*, as follows:

(1) $$R_{V}=R_{V}\left(0\right)-b\cdot d,$$

where *R_V_*(0) stands for the extrapolated value of the voltage responsivity at *d* = 0, and *b* is the slope of the approximation line. The voltage responsivity of the diodes DD0 is almost independent on the contact width, while the linear dependence of the voltage responsivity of the planar diodes on the base of AlGaAs is stronger, and the slope *b* increases with the increase in the AlAs mole fraction *x* in the semiconductor compound. It should be noted that the measurements of the detected voltage were performed using high impedance loading. If a 50 Ohms load was used, then the voltage responsivity would decrease. In the case of the DD15 diode, the responsivity decreases from 100 V/W down to 3 V/W. However, in the case of the 50 Ohms load, the DD15 diode can be used to detect pulsed nanosecond-long microwave signals. The electrical resistance *R*_0_ at zero voltage is scattered around its median value in the case of the DD0 and DD15 diodes, and it follows linear dependence close to Equation (1), with coefficient *b_R_* for the DD30 diodes. Table 2 summarizes the values of the voltage responsivity and electrical resistance of the planar microwave diodes in respect of the width of their narrow contact.

**Figure A2:**
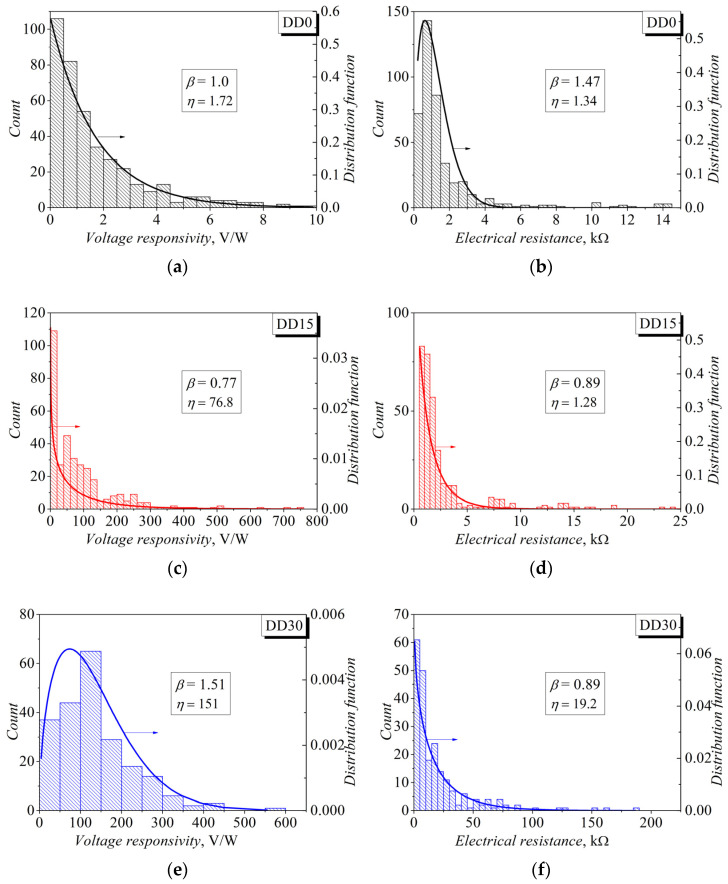
Histograms of the voltage responsivity (**a**,**c**,**e**) and electrical resistance (**b**,**d**,**f**) experimental data of the diodesw DD0 (**a**,**b**); DD15 (**c**,**d**); DD30 (**e**,**f**) approximated with Weibull distribution function.

We tried to approximate the statistical data of our experiments using the following various distribution functions: normal, lognormal, and Weibull. The last function was the best to fit the experimental results of the voltage responsivity and electrical resistance. Figure A2 in Appendix A depicts histograms of the experimental data of the voltage responsivity and electrical resistance of all three batches of the planar microwave diodes, DD0, DD15, and DD30. The solid lines in Figure A2 denote the Weibull distribution function. Its parameters, the shape *β* and the scale *η*, are presented in the legends of Figure A2 in Appendix A, and are summarized in Table 3.

A comparison of the data presented in Table 2 and Table 3 shows a qualitative correlation between the voltage responsivity and electrical resistance median data and scale parameter *η*, which is derived from the data approximation by the Weibull distribution function (Equation (A1) in Appendix A).

For a more detailed, further investigation, representative diodes having median values of the voltage responsivity *R_V_* and electrical resistance *R*_0_ were selected from all three batches. Electrical parameters of the selected planar microwave diodes measured under white-light illumination and in the dark are presented in Table 4.

**Figure 3:**
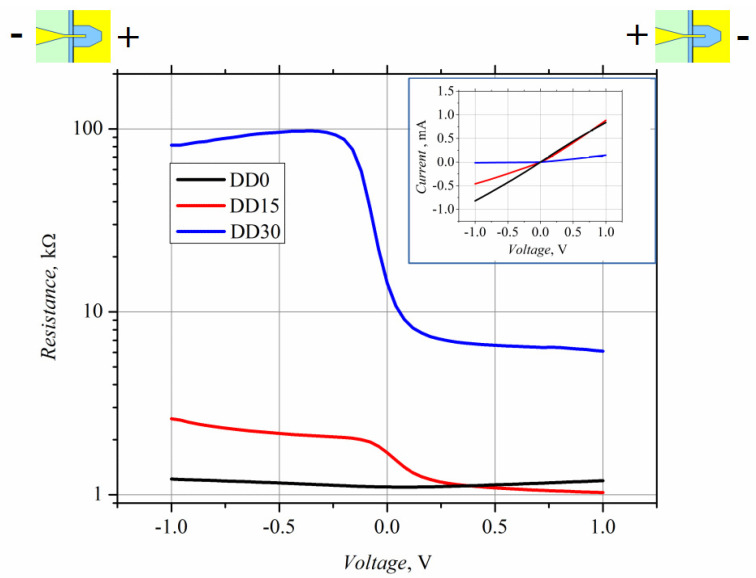
Dependence of electrical resistance of the illuminated planar microwave diodes on applied bias voltage. IV characteristics of the diodes are presented in the inset.

The IV characteristics of the white-light-illuminated diodes are presented in Figure 3. The forward current flows through the diodes when a positive potential of the voltage is applied to the small area contact, as is shown in Figure 3. The IV characteristic of the DD0 diodes is quasilinear, while it becomes nonlinear in the case of the DD15 and DD30 diodes. The nonlinearity is more strongly pronounced for the DD30 diode, which is fabricated on the base of higher resistivity Al_0.3_Ga_0.7_As semiconductor material. The asymmetry of the current–voltage characteristics also increases with the mole fraction *x*. Correlation of the IV characteristic’s asymmetry with the diode’s voltage responsivity allows us to expect an appropriate increase in responsivity as the diode demonstrates a more asymmetric IV characteristic. A reliable relation exists between the IV characteristic’s asymmetry and the diode’s voltage responsivity. When the relaxation of the average energy of hot electrons in a semiconductor can be neglected, and the conductivity current exceeds the displacement current, then the voltage responsivity of the microwave diode with *n-n^+^* junction can be expressed as [20].

(2) $$R_{V}=\frac{R_{r}-R_{f}}{2U},$$

where *R_r_* and *R_f_* denote the electrical resistance in the reverse and forward, respectively, direction of the applied bias voltage *U*.

**Figure 4:**
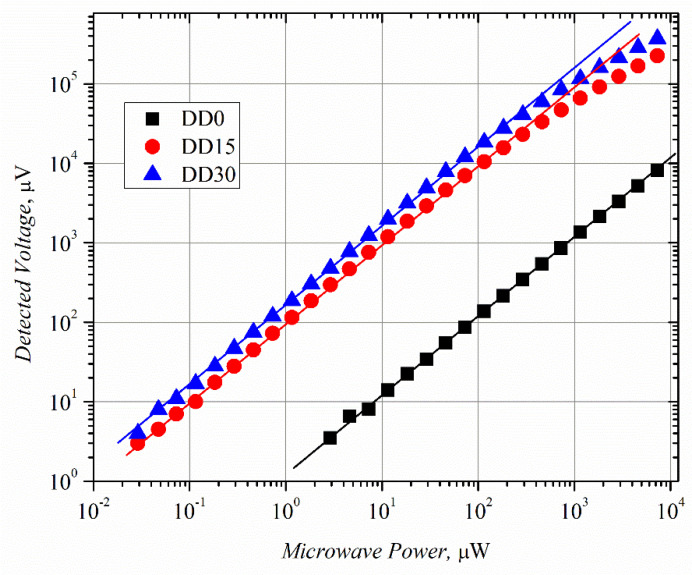
Voltage–power characteristics of the planar microwave diodes measured under white-light illumination at *f* = 30 GHz frequency. Lines are guides for the eye of linear dependence.

The dependence of the detected voltage on microwave power, i.e., VP characteristics of the planar microwave diodes, was measured using a high-frequency probe station at *f* = 30 GHz frequency. The characteristics are presented in Figure 4.

**Figure 5:**
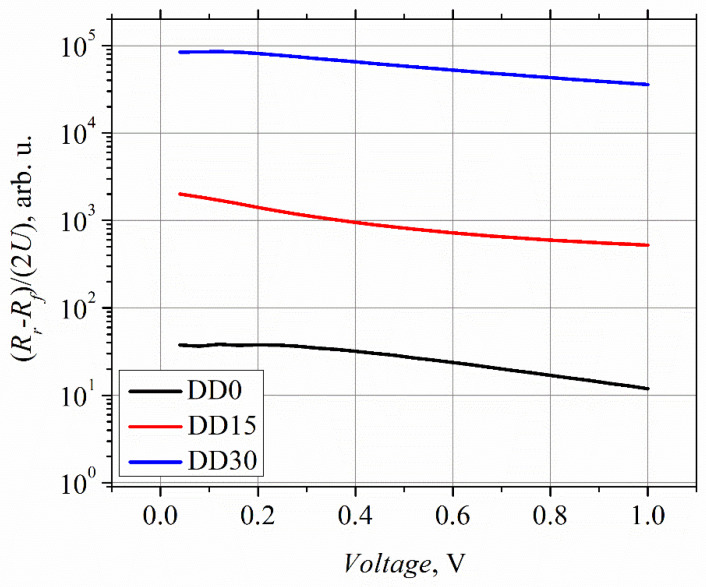
Dependence of the IV asymmetry of the planar microwave diodes on applied bias voltage.

The microwave power on the graph is the incident power (not the absorbed one), i.e., the power supplied to the HF probe. The voltage standing wave ratio (VSWR) was not measured in the case of the experiments carried out using the probe station. It can be conducted when the planar MW diode is mounted into a waveguide. At low MW power, the detected voltage of all the microwave diodes follows the linear law. However, the VP characteristics become different at higher power values; they turn sublinear in the case of the DD15 and DD30 diodes, while the DD0 diode’s characteristic remains linear, within the measured MW power range. The difference in the VP characteristics can be partly explained by the difference in the asymmetry of the IV characteristics. Figure 5 depicts the asymmetry versus applied voltage calculated using Equation (2).

The asymmetry of the DD0 diode’s IV characteristic is the lowest, and that of the DD15 diode is higher by almost two orders of magnitude. This correlates well with the VP experimental results (Figure 4), which found that the voltage responsivity of the DD15 diode is higher, by two orders of magnitude, as compared to that of the DD0 diode. On the other hand, the IV asymmetry of the DD30 diode is higher, by two orders of magnitude, than that of the DD15 diode, while the voltage responsivity is higher by only two times. Poor coincidence between the IV asymmetry and voltage responsivity data of the DD15 and DD30 diodes can be explained by weaker absorption of the incident MW radiation by the DD30 diode, due to the higher value of its electrical resistance (see Table 4). The deviation from the linear law (VP characteristics of diodes DD15 and DD30) most probably results from the decrease in the diodes’ IV asymmetry at a lower applied voltage than in the case of the DD0 diode.

As the data presented in Table 4 show, white-light illumination has a different impact on the electrical parameters of the microwave diodes. The DD0 diodes are more sensitive to the illumination; both their voltage responsivity and electrical resistance decrease by about 30% when the light is put on. The change in the parameters of the DD15 and DD30 diodes is lower, by about one order of magnitude, as compared to the DD0 diode. A weaker dependence of *R*_0_ on illumination is associated with the formation of different densities of activation energy levels at the metal–semiconductor interface, with an increase in the AlAs mole fraction *x* [32] and also with a different nature of deep traps in the bulk of the Al*_x_*Ga_1-*x*_As ternary semiconductor depending on *x* [33]. It is worth noting that the voltage responsivity of the diode DD30 increases with illumination. The IV asymmetry of the planar microwave diodes also correlates with the voltage responsivity, considering the impact of illumination; the light-induced change in the asymmetry decreases with the rise in *x* in the Al*_x_*Ga_1-*x*_As compound. This correlation also supports the presumption that the detected voltage arises due to the rectification of microwave currents across the quasi–ohmic metal–semiconductor junctions of the diodes.

Actually, the developed planar microwave diodes are a composition of two quasi-ohmic metal–semiconductor junctions. Therefore, a natural expectation is to describe the performance of the diodes using equations that are inherent to a metal–semiconductor junction. We fitted the experimental data of the IV characteristics of the diodes by the formula of a Schottky junction [34], as follows:

(3) $$I=I_{s}\left[\exp\left(\frac{eU}{nkT}\right)-1\right],$$

where *I* and *I_s_* are the current and the saturation current, respectively, *U* stands for the applied bias voltage, *n* marks the non-ideality factor, *k* notes the Boltzmann constant, and *T* is the diode temperature. The saturation current *I_s_* and the non-ideality factor *n* were chosen as the fitting parameters. The fitting was performed using IV data not exceeding 0.15 V of the applied voltage. The fitting parameters of the diodes are presented in Table 5.

The experimental IV characteristics of the diodes DD15 and DD30 only can be satisfactorily fitted using Equation (3). The best fitting results are achieved for the DD15 diodes, when the fitting parameters are scattered by ~4%, while the parameters of the diodes DD30 are scattered by ~10%. No satisfactory agreement between the IV experimental data and the approximation by Equation (3) was obtained for the DD0 diodes. An unusually high value of the non-ideality factor can be explained by the metal-semiconductor junction being far from the ideal Schottky junction. Ge/Ni/Au metals annealed at high temperatures make the quasi–ohmic metal-semiconductor junction have such a high value for the non-ideality factor.

If the IV characteristic of a microwave diode is described by Equation (3), then its voltage responsivity can be expressed as [35].

(4) $$R_{V}=\frac{\gamma R_{j}k_{abs}}{2\left(1+\frac{R_{s}}{R_{j}}\right)\left[1+\frac{R_{s}}{R_{j}}+{\left(\omega C_{j}\right)}^{2}R_{s}R_{j}\right]},$$

where $\gamma =\frac{d^{2}I}{dU^{2}}/\frac{dI}{dU}$ denotes the nonlinearity of the IV characteristic, *R_j_* and *C_j_* stand for the barrier resistance and capacitance of a metal–semiconductor junction, respectively, *R*_s_ is the series resistance of the diode, *ω* is the angular frequency of a microwave signal, and *k_abs_* notes the part of microwave radiation absorbed by the diode. Some parameters in the formula can be derived from the IV characteristic of the diode, namely, the non-linearity parameter *γ* and the electrical resistances of the diode. The barrier resistance *R_j_* can be calculated by means of subtracting the series resistance *R_s_* from the experimentally measured resistance *R*_0_, at zero applied voltage. However, in the case when the IV characteristic of the diode is far from the characteristic of an ideal Schottky junction (the case of the DD0 diode), the experimental estimation of *R_s_* becomes complicated. Therefore, we calculated the geometrical series resistance of the diode using the experimental value of the sheet resistance *R_sh_* of the epitaxial layer (see Table 1), with the following formula

(5) $$R_{s}=R_{sh}\frac{\left(D-d\right)ln\frac{D}{d}}{4Lln\frac{D}{d}+\pi \left(D-d\right)}.$$

**Figure 6:**
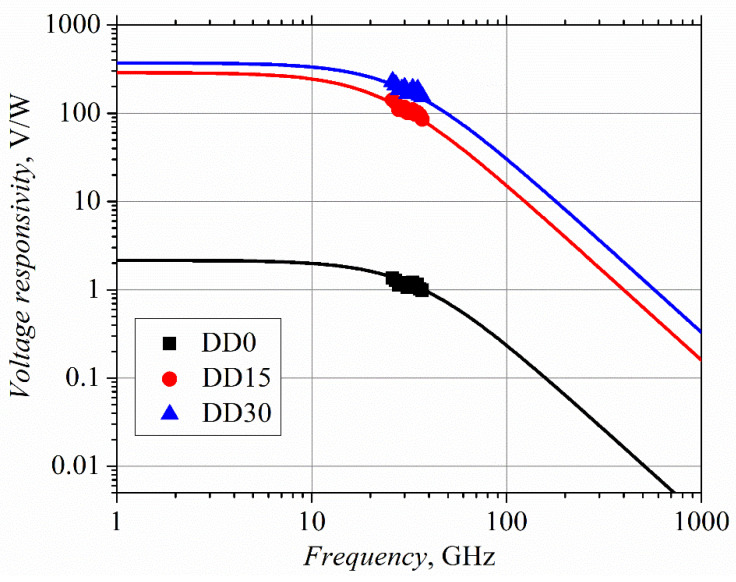
Experimental frequency dependence of voltage responsivity of the planar microwave diodes (dots) in the K_a_ frequency range. Solid lines mark the dependences in wider frequency range calculated using Equation (4).

Here, we used geometrical parameters of the diode *d, D* and *L*, which are shown in the inset of Figure 1. The series resistance of the diodes DD0, DD15 and DD30, calculated using Equation (5), was 190 Ω, 580 Ω, and 3460 Ω, respectively, taking the small contact width *d* = 1 μm, contact length *L =* 10 μm, and gap width *D* = 15 μm. Such evaluation of the series resistance of the diodes was only successful in the case of the DD30 diode. In the case of the DD15 and DD0 diodes, the calculated value was, respectively, 1.5 and 5 times lower than the experimental one. The mismatch between the calculated and experimental values can be associated with a stronger influence of the surface states, as the AlAs mole fraction in the semiconductor compound is reduced. The absorbed microwave power (absorption coefficient *k_abs_*), as well as the barrier capacitance *C_j_* of the diode can be found from Equation (4), and can be used to approximate the experimental dependence of the voltage responsivity of the planar microwave diodes on frequency, as is shown in Figure 6.

The parameters used to fit the calculated *R_V_* data to the experimental points are presented in Table 6.

Such an approximation technique opens the possibility of evaluating the high-frequency capacitance of the microwave diodes (as small as in the femtofarad range), as well as finding the microwave power absorbed in the diode. Moreover, the performance of the microwave diodes in the THz frequency range can also be forecasted. As Table 6 shows, the values of the voltage responsivity of the DD15 and DD30 diodes let us expect their application, even in the terahertz frequency range. Of course, our planar diodes are weak competitors with the Schottky diodes, in the sense of responsivity. However, the diodes having responsivity of the order of (0.1–1.0) V/W can be used to detect electromagnetic radiation in THz frequency range [21,23].

**Figure 7:**
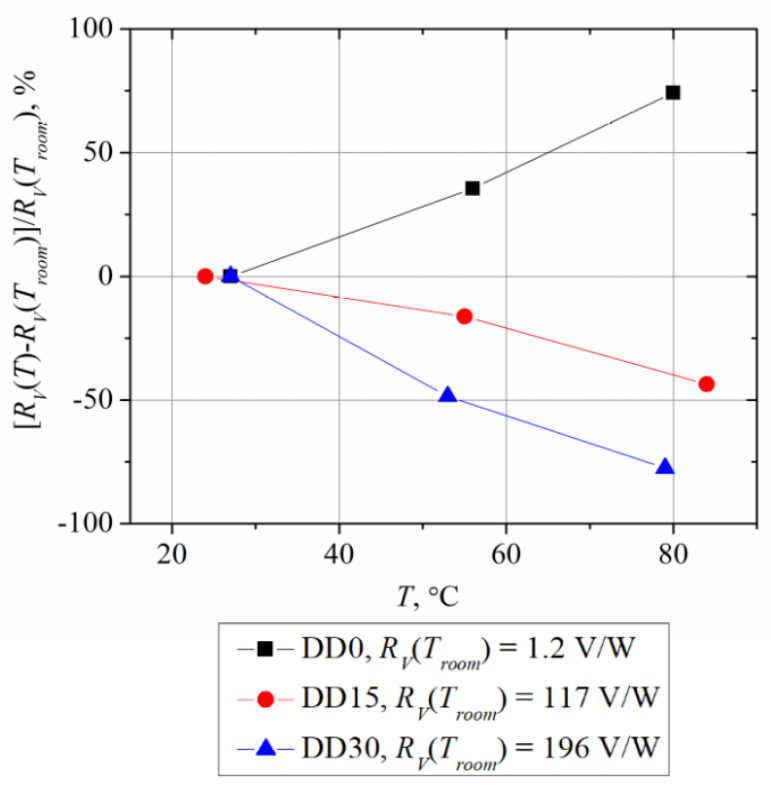
Temperature dependence of relative voltage responsivity change in the planar microwave diodes.

The relative change in voltage responsivity with temperature of all three types of diodes is depicted in Figure 7.

**Figure 8:**
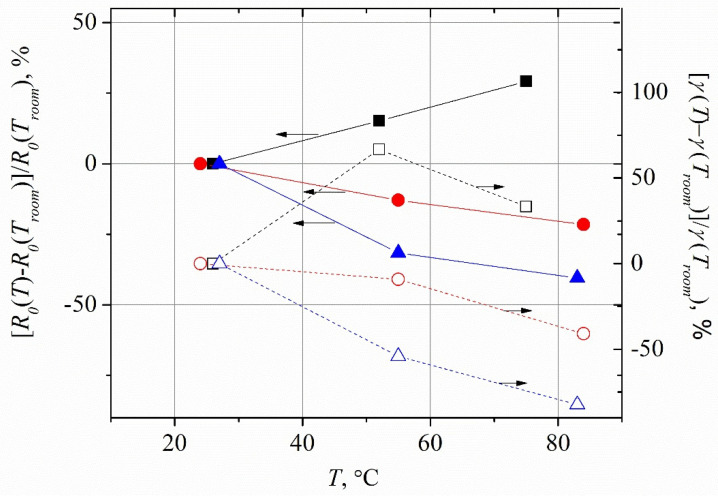
Temperature dependence of electrical resistance of the planar microwave diodes at zero applied voltage (solid dots), and temperature dependence of the non-linearity coefficient *γ* of the IV characteristics of the diodes at zero voltage (open dots). The colors correspond to the same type of the diodes as in Figure 7.

Since the planar microwave diode is composed of two metal–semiconductor junctions, connected in series in opposite directions (see Figure 1), this way, the resulting IV characteristic of such a planar diode should be made of backward branches of both junctions. Therefore, by following the equation below of the saturation current of a Schottky junction [34]:

(6) $$I_{s}=A_{R}T^{2}e^{-\frac{e\psi_{ms}}{kT}}$$

(here *A_R_* is the Richardson constant, *ψ_ms_* marks the barrier height of a Schottky junction), one should expect a decrease in electrical resistance of the planar microwave diode with higher temperatures. The temperature dependence of the electrical resistance of the diodes at zero applied voltage is presented in Figure 8, in solid points, and the values of the non-linearity coefficient *γ* of the IV characteristics are also depicted here, in open dots.

The electrical resistance of the diodes DD15 and DD30 drops down as their temperature rises. This characteristic agrees with saturation current dependence on temperature, as described by Equation (6). However, the dependence of the DD0 diode’s resistance on temperature is more stipulated by the bulk resistance of GaAs, and therefore the DD0 diode’s resistance increases with temperature. Moreover, the sign of the nonlinearity coefficient of its IV characteristic is also opposite to that of the diodes DD15 and DD30 (see Table 6). As Figure 7 and Figure 8 show, a good correlation exists between all three of the parameters, i.e., voltage responsivity, resistance, and nonlinearity coefficient, in their dependence on temperature. The detection properties of the diodes on the base of the Al*_x_*Ga_1-*x*_As ternary semiconductor (diodes DD15 and DD30) are determined by the rectification of microwave currents in the integrate planar dual microwave diode, which is composed of two metal–semiconductor quasi–ohmic junctions that are connected in series in opposite directions.

Finally, we carried out an evaluation of another important characteristic of a sensor. It is the ability of the microwave diode to withstand the impact of high-power microwave radiation. In the first step, we measured the maximum forward bias voltage (positive potential is applied to the small area contact), at which the non-reversible changes in the IV characteristic still do not take place. Then, the maximum electrical power absorbed by the diode is calculated and the maximum incident MW power is found, by taking the value of the absorption coefficient *k_abs_* from Table 6. The most robust planar microwave diode is DD30, which can withstand incident MW radiation of ~1 W without any changes in its electrical parameters. The diodes DD0 and DD15 were ten-fold weaker; their characteristics remain unchanged if the incident MW power does not exceed 100 mW.

A comparison of the electrical parameters of several millimeter wave zero voltage biased room-temperature diodes is presented in Table 7. Our planar diodes are not inferior to the other diodes, in the sense of the voltage responsivity at low radiation frequency. Moreover, though the voltage responsivity of the planar diodes decreases towards the higher frequencies, it remains sufficient to detect electromagnetic radiation in the sub-terahertz range. It should be emphasized, once again, that the planar diodes are attractive in their simplicity and do not require complex manufacturing technologies.

## 4. Conclusions

Summarizing the results of the research of planar microwave sensors on the base of *n*-Al_x_Ga_1-*x*_As having different AlAs mole fractions (*x* = 0, 0.15 and 0.3), we can draw the following conclusions:

1. Voltage responsivity of the aluminum containing *n*-Al*_x_*Ga_1-*x*_As sensors is by two orders of magnitude higher than that of the *n*-GaAs-based diodes;
2. Higher voltage responsivity of the planar microwave diodes on the base of *n*-Al*_x_*Ga_1-*x*_As is related to effective rectification of the microwave currents on the quasi–ohmic contacts of the diodes;
3. Voltage responsivity of the planar microwave diodes is sensitive to the white-light illumination; the lower the AlAs mole fraction in the *n*-Al_x_Ga_1-*x*_As diode, the stronger its sensitivity to the light;
4. The characteristics of voltage responsivity versus temperature depend on the *x* content in the sensor material. As temperature increases, the responsivity of the *n*-GaAs-based diodes increases, while it decreases in the case of aluminum-containing diodes; the diode with a higher *x* value is more temperature-sensitive. Different behavior of the sensors with temperature is related to a different quality of the quasi–ohmic metal–semiconductor contacts of the diodes; a higher AlAs mole fraction in the compound semiconductor stipulates higher asymmetry of the diode’s IV characteristic. As a result, the diodes with a higher *x* value behave more like metal–semiconductor Schottky junction diodes;
5. The sensors on the base of the *n*-Al_0.3_Ga_0.7_As compound can withstand incident microwave radiation power up to 1 W.

## Figures and Tables

**Table 1:** Parameters of the epitaxially grown Al*_x_*Ga_1-*x*_As layers.

AlAs Mole Fraction *x*	Layer Thickness *t*, μm	Electron Density *n*, cm^−3^	Electron Mobility *μ*, cm^2^/(V·s)	Electrical Resistivity ρ, Ω·cm	Sheet Resistance *R_sh_*, Ω/▯
0	0.8	5.5 × 10^16^	1820	6	780
0.15	0.8	2.0 × 10^16^	1680	19	2330
0.3	0.5	6.0 × 10^15^	1500	70	13,880

**Table 2:** Parameters of the linear dependence of voltage sensitivity and electrical resistance on diodes’ contact width.

Diode	Voltage Responsivity at *d* = 0 *R_V_*(0), V/W	Slope *b*, V/(W·μm)	Electrical Resistance at *d* = 0 *R*_0_(0), kΩ	Slope *b_R_*, kΩ/μm
DD0	0.9 ± 0.16	0.02 ± 0.06	1.0 ± 0.1	0.03 ± 0.04
DD15	98 ± 10	20.5 ± 3.9	2.16 ± 0.33	0.2 ± 0.12
DD30	207 ± 19	40 ± 7	15.6 ± 3.1	2.6 ± 1.2

**Table 3:** Shape and scale parameters of the Weibull distribution function for experimental voltage responsivity *R_V_* and electrical resistance *R*_0_ data.

Diode	Shape *β*		Scale *η*	
	for *R_V_*	for *R*_0_	for *R_V_*	for *R*_0_
DD0	1.0	1.47	1.72	1.34
DD15	0.77	0.89	76.8	1.28
DD30	1.51	0.89	151	19.2

**Table 4:** Voltage responsivity *R_V_* and electrical resistance *R*_0_ of the representative illuminated and darkened planar microwave diodes.

Diode	Illuminated		Dark	
	*R_V_*, V/W	*R*_0_, kΩ	*R_V_*, V/W	*R*_0_, kΩ
DD0	1.2	1.1	1.7	1.8
DD15	100	1.70	105	1.8
DD30	160	14.3	150	14.9

**Table 5:** Fitting parameters of the IV characteristics approximated with Equation (3).

Diode	Saturation Current *I_s_*, A	Non-Ideality Factor *n*
DD0	(3.5 ± 2.1) × 10^−2^	1520 ± 15
DD15	(2.9 ± 0.12) × 10^−4^	20 ± 0.7
DD30	(2.3 ± 0.24) × 10^−5^	11.2 ± 0.9

**Table 6:** Experimental and approximation parameters of the planar microwave diodes.

Diode	*γ*, V^−1^	*R_j_*, kΩ	*R_s_*, kΩ	*C_j_*, fF	*k_abs_*,%	*R_V_* (1 GHz), V/W	*R_V_* (1 THz), V/W
	Experimental Parameters			Approximation Parameters		Approximated Parameters	
DD0	0.07	0.9	0.19	12	10	1.8	0.003
DD15	2.2	1.23	0.54	10	44	290	0.16
DD30	8.5	10.9	3.6	1	1.4	370	0.35

**Table 7:** Parameters of several different millimeter wave diodes operating at room temperature without bias voltage.

Diode	Technology	Dimension of Active Part	*R*_0_, kΩ	*R_v_*_at 30 GHz_, V/W	*R_v_*_at 1 THz_, V/W	Reference
Planar DD15	LPE	1 μm	1.7	100	0.17	This paper
Planar DD30	LPE	1 μm	14.3	160	0.33	This paper
Bow-tie GaAs	VPE	10 μm	4.0	0.3	0.1	[21]
Gated bow-tie GaAs TEMF	MBE	2 μm	5.5	4.0	2.0	[24]
Gated bow-tie GaAs Schottky	MBE	2 μm	5.5	70	0.06	[24]
Heterojunction	MBE	2 μm	1.0	300	0.4	[26]
Bow-tie InGaAs	MBE	12 μm	9.3	5.0	2.0	[36]
Self-switching (SSD)	MOCVD	90 nm	1.5	80	2.0	[37]
Graphene ballistic rectifier	Graphene/Boron nitride	500 nm	6.0	800	0.05	[38]

## Appendix A

Statistical presentation of the voltage responsivity and electrical resistance of the planar microwave diodes.

The statistical representation was executed by testing diodes in batches each containing 480 units. The box-charts present data of the diodes on the base of GaAs (diodes DD0), on the base of Al_0.15_Ga_0.85_As (diodes DD15) and on the base of Al_0.3_Ga_0.7_As (diodes DD30) shown in Figure A1 (a, b), (c, d) and (e, f), respectively. Outputs of the fabricated planar diodes in the respective DD0, DD15 and DD30 batches are 83%; 78% and 46%.

Experimental data of the voltage responsivity and electrical resistance of the planar microwave diodes is approximated using Weibull distribution function [39]

(A1) $$f\left(x\right)=\frac{\beta }{\eta }{\left(\frac{x}{\eta }\right)}^{\beta -1}\exp\left[-{\left(\frac{x}{\eta }\right)}^{\beta }\right],$$

where *x* stands for either voltage responsivity or electrical resistance of the planar microwave diode, *β* and *η* mark the shape and the scale parameters of the Weibull distribution, respectively. The histograms of the voltage responsivity and electrical resistance of the planar diodes are presented in Figure A2.
